# Excellent agreement of Norwegian trauma registry data compared to corresponding data in electronic patient records

**DOI:** 10.1186/s13049-023-01118-5

**Published:** 2023-09-26

**Authors:** N Naberezhneva, Oddvar Uleberg, M Dahlhaug, V Giil-Jensen, K G Ringdal, O Røise

**Affiliations:** 1 Biobank and Registry Support Department, Division for medical quality registries for South- Eastern Norway Regional Health Authority, Oslo University Hospital, Oslo, Norway; 2 Department of Research and Development, Division of Emergencies and Critical Care, Oslo University Hospital, Oslo, Norway; 3 Department of Emergency Medicine and Pre-hospital services, St. Olav`s University Hospital, Trondheim, Norway; 4 Norwegian Trauma Registry, Division of Orthopedics, Oslo University Hospital, Oslo, Norway; 5 Western Norway Trauma Center, Haukeland University Hospital, Bergen, Norway; 6 Department of Anesthesiology, Vestfold Hospital Trust, Tønsberg, Norway; 7 Division of Prehospital Care, Vestfold Hospital Trust, Tønsberg, Norway; 8 Institute of Clinical Medicine, Faculty of Medicine, University of Oslo, Oslo, Norway

**Keywords:** Data Quality, Data Accuracy, Data Collection, Health Care Evaluation Mechanisms, Wounds and Injuries, Registries

## Abstract

**Background:**

The Norwegian Trauma Registry (NTR) is designed to monitor and improve the quality and outcome of trauma care delivered by Norwegian trauma hospitals. Patient care is evaluated through specific quality indicators, which are constructed of variables reported to the registry by certified registrars. Having high-quality data recorded in the registry is essential for the validity, trust and use of data. This study aims to perform a data quality check of a subset of core data elements in the registry by assessing agreement between data in the NTR and corresponding data in electronic patient records (EPRs).

**Methods:**

We validated 49 of the 118 variables registered in the NTR by comparing those with the corresponding ones in electronic patient records for 180 patients with a trauma diagnosis admitted in 2019 at eight public hospitals. Agreement was quantified by calculating observed agreement, Cohen’s Kappa and Gwet’s first agreement coefficient (AC_1_) with 95% confidence intervals (CIs) for 27 nominal variables, quadratic weighted Cohen’s Kappa and Gwet’s second agreement coefficient (AC_2_) for five ordinal variables. For nine continuous, one date and seven time variables, we calculated intraclass correlation coefficient (ICC).

**Results:**

Almost perfect agreement (AC_1_ /AC_2_/ ICC > 0.80) was observed for all examined variables. Nominal and ordinal variables showed Gwet’s agreement coefficients ranging from 0.85 (95% CI: 0.79–0.91) to 1.00 (95% CI: 1.00–1.00). For continuous and time variables there were detected high values of intraclass correlation coefficients (ICC) between 0.88 (95% CI: 0.83–0.91) and 1.00 (CI 95%: 1.00–1.00). While missing values in both the NTR and EPRs were in general negligeable, we found a substantial amount of missing registrations for a continuous “Base excess” in the NTR. For some of the time variables missing values both in the NTR and EPRs were high.

**Conclusion:**

All tested variables in the Norwegian Trauma Registry displayed excellent agreement with the corresponding variables in electronic patient records. Variables in the registry that showed missing data need further examination.

**Supplementary Information:**

The online version contains supplementary material available at 10.1186/s13049-023-01118-5.

## Background

An important part of a mature and inclusive trauma system is a reliable trauma registry with high-quality data in order to deliver better therapeutic options and care that is more efficient and with reduced morbidity and mortality [1–3]. In addition, data from registries with uniform reporting of variables can provide benchmark data, which allows for comparisons between patients, institutions, regions and countries [4, 5]. Continuous quality measurement of health services is becoming increasingly important on the path towards value-based health care [6, 7] and data from medical quality registries are more frequently used as a source when forming public health policy [8].

However, it is essential that registered and reported data are as accurate and complete as possible, since unreliable registrations can cause misleading statistics at both regional and national levels [9, 10]. Several studies have examined the quality of trauma registry data and found major limitations in both data quality and completeness [9, 11, 12], missing data [13, 14] and simple inconsistencies and misinterpretations of clinical notes in electronic patient records [15]. The fact that many studies focus solely on injury coding variables [3, 15–17] and not on the majority of variables registered, can be seen as a limitation to the broader assessment of data quality in trauma registries. Lack of continuous monitoring and validation processes of the quality of trauma registry data has also been underlined as a potential cause of reduced validity and reliability [4].

Data quality assessment in trauma registries is challenging as there is currently no international agreement on classification of data quality dimensions, measurement techniques and how to improve data quality [17]. According to Wang and Strong`s conceptual model for analyzing and improving health care data, data quality can be measured in six dimensions – completeness, accuracy, precision, correctness, consistency and timelines [12, 18]. Arts et al. described that the two most cited data quality measures are accuracy (the extent to which registered data are in conformity to the truth, e.g. patient records) and completeness (the extent to which all necessary data that could have been registered have actually been registered) [17, 19].

The Norwegian Trauma Registry (NTR) is a national medical quality registry that includes data from all trauma-receiving hospitals in Norway. The NTR dataset is based on, but includes more data, than the revised Utstein Template for Uniform Reporting of Data Following Major Trauma [5]. The objective of this study was to assess the accuracy of data in the NTR by comparing registry data to corresponding data in electronic patient records (EPRs) in a sample of 180 patients treated at eight of the 38 Norwegian trauma receiving hospitals.

## Methods

### The Norwegian trauma registry (NTR)

The NTR is one of the 59 national medical quality registries (2023) in Norway where all hospitals receiving and treating seriously or potentially seriously injured patients are required by national regulations to submit data [20, 21]. In 2019, all 38 Norwegian hospitals (34 acute care hospitals and 4 trauma centers) reported to the NTR. The NTR has certified registrars (data coders) at each hospital. Patients who satisfy the inclusion criteria are entered into the registry without consent but can actively opt out. All patients who are received by a trauma-team are included. In addition, all hospitals are obliged to search for admitted patients with a New Injury Severity Score (NISS) > 12 (which indicates severe injury) that were not received by a trauma team. The registry collects clinical data on about 9.000 patients per year with full hospital coverage level. For patients who were received with a multidisciplinary trauma-team, the patient coverage level in the registry is 92.2% [22].

The NTR uses a national electronic medical registration solution (MRS), which allows local hospital databases to function as local quality registries and export data to the national registration solution. In 2019, the NTR personnel collected 118 variables (of which 35 were Utstein variables) [5], describing the trauma period, accident information, pre-hospital data, emergency data, hospital data, injury scoring and result data, from the emergency scene throughout the chain of acute care including measures of rehabilitation.

Continuous internal data quality assurance is of high priority and several steps are taken to warrant that high-quality data are entered into the registry. There are three data validation mechanisms built into the MRS: (1) all personal identification entries are automatically checked against the National Population Registry, (2) validation mechanisms that detect evident data outliers and (3) registration forms without entries of compulsory data fields are not possible to submit. In order to secure uniform understanding for the registrars, the NTR has developed a dictionary in native language defining known difficult medical terms in the Abbreviated Injury Scale (AIS) dictionary. A data definition catalogue with description of all variables (e.g. variable definition, type, category, values, fieldname, and coding explanation) has been presented [23] and is annually revised to reduce inconsistencies. In addition, the NTR Secretariat provides continuous support to hospital registrars through guidelines, information letters and user-support by e-mail and telephone [24]. All the national medical quality registries and health registries in Norway are obliged to measure the quality of data recorded. The NTR has a rolling plan over five years for data quality assessments at each hospital.

### Data collection

Four regional health authorities (RHA) are responsible for the 38 trauma-receiving hospitals in Norway, where each RHA has one trauma center and several acute care hospitals. In this study, one high-volume acute care hospital, three regional trauma centers and four low-volume care hospitals (one from each RHA) were selected to provide a representative sample of Norwegian hospitals. We included all the patients who were registered in the NTR during one study month in 2019 (a total of 198 patients), 16 of those were transferred from other hospitals and two patients were not trauma patients. Those cases were excluded and the sample size was thus reduced to 180 patients. The one month study period (May 2019) was chosen to ensure a high likelihood of finalized reporting into the NTR, as there is known to be a delay of several months (e.g. due to patient length of stay, 30-day outcome measure and capacity of registrars). Moreover, this month represents on average a 10% caseload of annual patients registered into the NTR [24]. Forty-nine variables (18 of which are Utstein template variables) had been selected before the data collection started. These variables are included in the registry’s main quality indicators (system, process and outcome indicators), and are important for research purposes. In addition, variables that were considered difficult to register, according to the NTR Secretariat, were selected (Additional File 1). Two experienced and certified registrars (authors MD and VG-J) performed the on-site data quality audits, together with the local hospital registrar. This audit team was blinded to the data already collected in the NTR. The team made a renewed registration of the data from the patients´ EPRs, compared these data with the data on the same patients previously registered in the NTR and noted correctness (yes/no).

### Statistical analysis

The goodness-of-fit approach by Donner and Eliasziw [25] says that when testing for a statistical difference between moderate (0.40) and excellent (0.90) Cohen’s Kappa values, based on alpha (0.05) and beta 0.1 error rates, sample size estimates range from 13 to 66 [26–29]. This recommended sample size calculation for the Kappa statistic was used in determining the sample size in the current study. Our sample of 180 trauma patients contained the required numbers to detect robust estimates of inter-rater reliability and was consequently deemed appropriate. Narrow confidence intervals of the results also confirm that the sample size was adequate.

To assess the data quality in the NTR, we quantified the agreement between the NTR and EPRs by calculating observed agreement, and both Cohen’s Kappa and Gwet’s AC_1_ (the first-order agreement coefficient) with 95% confidence intervals for nominal variables. For ordinal variables, we used the quadratic weighted Cohen’s Kappa and Gwet’s AC_2_ (the second-order agreement coefficient). The response category “unknown” was included for nominal variables but excluded for ordinal variables. American Society of Anesthesiologists physical status (ASA), Glasgow Outcome Score (GOS) and Glasgow Coma Scale (GCS) score were analyzed as ordinal variables in the agreement analysis.

For continuous, date and time variables, we calculated the intraclass correlation coefficient (ICC) with 95% confidence intervals using a two-way random effects model with type of absolute agreement [30, 31]. The mean and the standard deviation of the differences between the NTR and EPR registrations were measured to reveal the magnitude of disagreement. We converted time variables into decimal numbers (minutes after midnight) in Excel when the corresponding date variable was the same for both data sources [32]. The trauma date variable was formatted as number of days after December 31, 2018 [33]. Injury Severity Score (ISS) and NISS classifications were analyzed as continuous variables. Missing data in one or the other data source for any type of variables were excluded.

Cohen’s Kappa, Gwet’s AC_1_/AC_2_ and ICC with values ≤ 0.20 are interpreted as slight agreement, 0.21–0.40 as fair agreement, 0.41–0.60 as moderate agreement, 0.61–0.80 as substantial agreement, and values above 0.80 as almost perfect agreement [34].

Cohen’s Kappa statistic – a chance-corrected agreement measure – can be very sensitive to trait prevalence in the subject population. It can be particularly unstable and difficult to interpret in situations where a large proportion of the ratings are either positive or negative. The variable in question will then exhibit what is further specified as a skewed trait distribution, which, in turn, affects the Kappa statistic and leads to an artificially reduced Kappa coefficient [35]. Gwet’s AC_1_/AC_2_ are not influenced by trait prevalence [35, 36]. Hence, agreement was interpreted based on Gwet’s AC_1_/AC_2_ and observed agreement for variables with substantial discrepancies between the Kappa and AC_1_/AC_2_ coefficients, where the Kappa coefficient was considered artificially low due to a skewed trait prevalence. Distribution of trait prevalence for all variables is shown in Additional File 2.

Data were analyzed using STATA/SE 17.0 for Windows. For Cohen’s Kappa, Gwet’s AC_1_/AC_2_, we run the «kappaetc» function in STATA [37].

## Results

The overall results for categorical data (nominal and ordinal variables) are summarized in Tables 1 and 2. Out of 32 categorical variables, 28 (88%) variables showed excellent agreement with Gwet’s AC_1_/AC_2_ > 0.95. For the remaining four variables (“Helmet use”, “Mechanism of Injury (MOI) – fall”, “Pre-hospital care level” and “Hospital care level”) we also discovered high Gwet’s first-order agreement coefficients of 0.87 (95% CI: 0.81–0.93), 0.93 (95% CI: 0.88–0.98), 0.85 (95% CI: 0.79–0.91), and 0.89 (95% CI: 0.84–0.94) respectively.

While all of the categorical variables displayed high percentages of observed agreement (87–100%) and high Gwet’s AC_1_/AC_2_ coefficients (0.85–1.00), four of those demonstrated corresponding Kappa values between 0.66 and 0.77 indicating substantial level of agreement 0.66 (95% CI: 0.04–1.00) for “Pre-hospital decompression”; 0.71 (95% CI: 0.61–0.82) for “Pre-hospital care level”; 0.77 (95% CI: 0.67–0.88) for “Pre-injury ASA”; 0.77 (95% CI: 0.64–0.89) for “Discharge GOS”), and two variables (“Pre-injury GOS” and “Trauma team”) showed Kappa values of -0.01 (95% CI: -0.02–0.00) and 0.00 (95% CI: 0.00–0.00) respectively.

**Table 1:** Observed agreement, Cohen’s Kappa and Gwet’s AC_1_ for nominal variables

Variable	Patients (n)	Number of missing registrations		Number of registrations^a^	Observed agreement		Kappa (95% CI)	AC_1_ (95% CI)
		NTR	EPR		Observed agreement (%)^b^	Conc.(N); non-conc. (N)^c^		
***Accident and injury related data***:								
Helmet use	180	0	2	178	89.9	160; 18	0.84 (0.77–0.91)	0.87 (0.81–0.93)
MOI transport	180	0	0	180	96.1	173; 7	0.92 (0.87–0.98)	0.95 (0.91–0.99)
MOI road traffic	180	0	0	180	97.2	175; 5	0.95 (0.90–0.99)	0.96 (0.93-1.00)
MOI road traffic type	93	5	1	87	96.6	84; 3	0.95 (0.89-1.00)	0.96 (0.92-1.00)
MOI road traffic role	93	5	1	87	97.7	85; 2	0.91 (0.78-1.00)	0.98 (0.94-1.00)
MOI fall	180	0	0	180	96.1	173; 7	0.91 (0.84–0.98)	0.93 (0.88–0.98)
MOI violence	180	0	0	180	100.0	180; 0	1.00 (1.00–1.00)	1.00 (1.00–1.00)
MOI self inflicted	180	0	0	180	100.0	180; 0	1.00 (1.00–1.00)	1.00 (1.00–1.00)
MOI work accident	180	0	0	180	98.9	178; 2	0.95 (0.88-1.00)	0.99 (0.97-1.00)
MOI sports	180	0	0	180	98.9	178; 2	0.95 (0.88-1.00)	0.99 (0.97-1.00)
MOI fire	180	0	0	180	100.0	180; 0	1.00 (1.00–1.00)	1.00 (1.00–1.00)
MOI other	180	0	0	180	97.8	176; 4	0.88 (0.76-1.00)	0.97 (0.95-1.00)
MOI	180	0	0	180	96.1	173; 7	0.95 (0.92–0.99)	0.96 (0.93–0.99)
Injury intention	180	0	0	180	100.0	180; 0	1.00 (1.00–1.00)	1.00 (1.00–1.00)
Blunt injury	180	0	0	180	98.3	177; 3	0.89 (0.76-1.00)	0.98 (0.96-1.00)
Penetrating injury	180	0	0	180	100.0	180; 0	1.00 (1.00–1.00)	1.00 (1.00–1.00)
Dominating injury	180	0	0	180	99.4	179; 1	0.97 (0.91-1.00)	0.99 (0.98-1.00)
***Prehospital data***:								
Pre-hospital airway	173	1	1	172	99.4	171; 1	0.91 (0.72-1.00)	0.99 (0.98-1.00)
Pre-hospital decompression	173	1	1	172	99.4	171; 1	0.66 (0.04-1.00)	0.99 (0.98-1.00)
Pre-hospital care level	172	1	1	171	86.5	148; 23	0.71 (0.61–0.82)	0.85 (0.79–0.91)
Hospital type	175	1	1	174	97.7	170; 4	0.90 (0.81-1.00)	0.97 (0.95-1.00)
***Emergency admission data***:								
Trauma team	180	0	0	180	99.4	179; 1	0.00 (0.00–0.00)	0.99 (0.98-1.00)
In-hospital intubation	180	0	0	180	99.4	179; 1	0.95 (0.84-1.00)	0.99 (0.98-1.00)
In-hospital chest drainage	180	0	0	180	100.0	180; 0	1.00 (1.00–1.00)	1.00 (1.00–1.00)
Emergency intervention	180	0	0	180	100.0	180; 0	1.00 (1.00–1.00)	1.00 (1.00–1.00)
***Hospital stay data***:								
Hospital care level	180	0	0	180	91.1	164; 16	0.87 (0.81–0.93)	0.89 (0.84–0.94)
Discharge destination	179	0	0	179	96.6	173; 6	0.90 (0.83–0.98)	0.96 (0.94–0.99)

**Notes**:

^a^Number of cases for registrations present in both NTR and EPRs;

^b^Observed agreement calculated as number of concordant answers divided by *n* and multiplied by 100%;

^c^Concordant answers – agreements over all paired answers for a given variable.

**Abbreviations**:

NTR, Norwegian Trauma Registry; EPR, Electronic Patient Record; Conc., concordant answers; non-conc., non-concordant answers; AC_1_, Gwet’s first agreement coefficient; CI, confidence interval; MOI, Mechanism of Injury

**Table 2:** Observed agreement, Cohen’s quadratic weighted Kappa and Gwet’s AC_2_ for ordinal variables

Variable	Patients (n)	Number of missing registrations		Number of registrations^a^	Observed agreement (%)^b^	Kappa^c^ (95% CI)	AC_2_ (95% CI)
		NTR	EPR				
***Preinjury status data***:							
Pre-injury GOS	180	0	1	179	98.3	-0.01 (-0.02-0.00)	0.98 (0.96-1.00)
Pre-injury ASA	180	0	0	180	97.9	0.77 (0.67–0.88)	0.96 (0.94–0.98)
***Prehospital data***:							
Pre-hospital GCS	173	7	8	162	99.8	0.96 (0.91-1.00)	1.00 (0.99-1.00)
***Emergency admission data***:							
In-hospital GCS	180	0	0	180	99.4	0.85 (0.60-1.00)	0.99 (0.98-1.00)
***Hospital stay data***:							
Discharge GOS	180	0	4	176	98.7	0.77 (0.64–0.89)	0.97 (0.96–0.98)

**Notes**:

^a^Number of cases for registrations present in both NTR and EPRs;

^b^Quadratic weighted observed agreement;

^c^Cohen’s quadratic weighted Kappa, the category “unknown” is excluded.

**Abbreviations**: NTR, Norwegian Trauma Registry; EPR, Electronic Patient Record; AC_2_, Gwet’s second agreement coefficient; CI, confidence interval; GOS, Glasgow Outcome Score; ASA, American Society of Anesthesiologists physical status; GCS, Glasgow Coma Scale.

Tables 3 and 4 show that for registrations present in both the NTR and EPRs, excellent agreement with ICC ranging from 0.88 (95% CI: 0.83–0.91) to 1.00 was identified for all continuous, date and time variables. The mean difference and variance between the two data sources were bigger for “Pre-hospital Systolic Blood Pressure (SBP)”, “Injury date” and “Time scene arrival” than for the other above-mentioned variables.

**Table 3:** Intraclass correlation coefficient (ICC) for continuous variables

Variable	Patients (n)	Number of missing registrations		Number of registrations^a^	Observed agreement		ICC^d^ (95% CI)	Mean diff.^e^	SD^f^
		NTR	EPR		Observed agreement (%)^b^	Conc.(N); non-conc. (N)^c^			
***Prehospital data***:									
Pre-hospital SBP	150	14	5	136	86.8	118; 18	0.88 (0.83–0.91)	2.76	12.57
Pre-hospital RR	140	12	7	127	96.1	122; 5	0.99 (0.98–0.99)	0.02	0.94
***Emergency admission data***:									
In-hospital SBP	172	4	2	168	91.7	154; 14	0.99 (0.98–0.99)	-0.11	3.44
In-hospital RR	169	5	4	163	96.3	157; 6	0.99 (0.99-1.00)	-0.10	0.66
Base excess	91	19	1	71	100.0	71; 0	1.00 (.,. )	0.00	0.00
***Hospital stay data***:									
LOS ICU	180	0	0	180	90.0	162; 18	0.89 (0.85–0.91)	-0.11	0.52
Ventilator days	180	0	0	180	98.3	177; 3	0.98 (0.97–0.99)	0.01	0.13
***Injury scoring data***:									
ISS	180	0	0	180	87.2	157; 23	0.96 (0.94–0.97)	0.07	2.13
NISS	180	0	0	180	86.1	155; 25	0.96 (0.94–0.97)	0.26	2.58

**Notes**:

^a^Number of cases for registrations present in both NTR and EPRs;

^b^Observed agreement calculated as number of concordant answers divided by *n* and multiplied by 100%;

^c^Concordant answers – agreements over all paired answers for a given variable;

^d^Intraclass correlation coefficient (two-way random effects model with absolute agreement);

^e^Mean difference calculated as EPRs minus NTR;

^f^Standard deviation of the difference.

**Abbreviations**: NTR, Norwegian Trauma Registry; EPR, Electronic Patient Record; Conc., concordant answers; non-conc., non-concordant answers; ICC, intraclass correlation coefficient; CI, confidence interval; SD, standard deviation; SBP, Systolic Blood Pressure; RR, Respiratory Rate; LOS, Length of Stay; ICU, Intensive Care Unit; ISS, Injury Severity Score; NISS, New Injury Severity Score

Completeness of registrations was lower for “Pre-hospital SBP”, “Pre-hospital Respiratory Rate (RR)”, “In-hospital SBP” and “In-hospital RR”, Table 3. The medical procedure was not accomplished for 49% of the total number of examined patients for “Base excess” (n = 91). The same applies to “Time until chest x-ray” (14.4% not accomplished; n = 154), “Time until pelvic x-ray” (27.8%; n = 130) and “Time until first Computer Tomography (CT)” (22.8%; n = 139), Table 4.

A substantial amount of missing values was observed for “Base excess” (20.9% in the NTR; 1.1% in EPRs). “Pre-hospital SBP” variable had 9.3% of missing registrations in the NTR and 3.3% in EPRs. “Pre-hospital RR” had missing data of 8.6% in the NTR and 5.0% in EPRs.

**Table 4:** Intraclass correlation coefficient (ICC) for date and time variables

Variable	Patients (n)	Number of missing registrations		Number of registrations^a^	Observed agreement		ICC^d^ (95% CI)	Mean diff.^e^	SD^f^
		NTR	EPR		Observed agreement (%)^b^	Conc.(N); non-conc. (N)^c^			
***Accident time***:									
Trauma date^g^	180	0	0	180	99.4	179; 1	1.000 (1.000–1.000)	-0.01	0.07
***Accident and injury related data***:									
Injury date^h^	180	0	7	173	87.9	152; 21	0.9973 (0.9963–0.9980)	2.82	21.92
***Prehospital data***:									
Time scene arrival^h^	173	12	13	158	88.6	140; 18	0.9992 (0.9989–0.9994)	-2.46	12.08
Time scene departure^h^	173	14	14	156	90.4	141; 15	0.9984 (0.9979–0.9989)	0.20	17.30
***Emergency admission data***:									
Hospital arrival^h^	180	1	3	176	89.2	157; 19	1.0000 (1.0000–1.0000)	-0.01	2.07
Time until chest x-ray^h^	154	13	13	139	95.7	133; 6	1.0000 (1.0000–1.0000)	-0.04	1.41
Time until pelvic x-ray^h^	130	14	13	114	97.4	111; 3	1.0000 (1.0000–1.0000)	-0.15	1.51
Time until first CT^h^	139	33	34	103	76.7	79; 24	0.9999 (0.9999-1.0000)	0.12	3.93

**Notes**:

^a^Number of cases for registrations present in both NTR and EPRs;

^b^Observed agreement calculated as number of concordant answers divided by *n* and multiplied by 100%;

^c^Concordant answers – agreements over all paired answers for a given variable;

^d^Intraclass correlation coefficient (two-way random effects model with absolute agreement);

^e^Mean difference calculated as EPRs minus NTR;

^f^Standard deviation of the difference;

^g^Date variable was recalculated as the number of days after December 31, 2018;

^h^Time variables with the same date in both NTR and EPRs were recalculated as minutes past midnight.

**Abbreviations**: NTR, Norwegian Trauma Registry; EPR, Electronic Patient Record; Conc., concordant answers; non-conc., non-concordant answers; ICC, intraclass correlation coefficient; CI, confidence interval; SD, standard deviation; CT, Computer Tomography

Time variables showed missing values in both the NTR and EPRs with highest proportions of missing registrations for “Time until first CT” (23.7% in the NTR and 24.5% in EPRs) and “Time until pelvic x-ray” (10.8% in the NTR and 10.0% in EPRs), following “Time until chest x-ray” (8.4% in both data sources) and “Time scene departure” (8.1% in both data sources).

## Discussion

High-quality data in medical quality registries are imperative to ensure that the extracted information can be trusted and that it reflects the real world. In this study, we performed an internal audit of the NTR to determine whether the data in the registry are trustworthy. By comparing the registry data with EPRs we discovered that data accuracy of the NTR is excellent, even though there are some variables with reduced completeness that needs to be addressed.

The results showed substantial discrepancies between the Kappa and Gwet’s AC_1_/AC_2_ coefficients for three variables. We perceived Kappa values as being artificially low due to a skewed trait distribution for “Pre-hospital decompression”, and extremely skewed trait distribution for “Pre-injury GOS” and “Trauma team” (Additional File 2). With low Kappa despite high observed agreement and high Gwet’s coefficients, the agreement was thus considered almost perfect for these variables. When it comes to “Pre-hospital care level”, “Pre-injury ASA” and “Discharge GOS” having high Gwet’s coefficients and corresponding Kappa values within substantial level of agreement, the concordance was deemed substantial to almost perfect for those variables (reflected also in the lower and upper bounds of confidence intervals for respective Kappa values). Lower observed agreement was detected for almost all continuous, date and time variables, as compared with their ICCs, within the same agreement classification boundaries. However, the variable “Time until first CT” with 76.7% in observed agreement implying substantial level of agreement deviated considerably from its perfect ICC of 1.00. The possible explanation for this difference can be the fact that ICC analyzes the consistency of concordance allowing close numerical values to be concordant even though they are different. When using observed agreement, we just compare two numbers and assign a binary classification of equal or different.

### Comparability

Few studies have evaluated the full extent of data quality of trauma registries, and these often differ in study inclusion criteria, examined variables and inclusion criteria of registry itself. European trauma registries such as the Trauma Register DGU [38], The Trauma Audit & research Network (TARN) [39] and the Swedish Trauma Registry [40], alongside with the NTR, all use different registry inclusion criteria. In a recent study by Holmberg et al., for example, the authors underlined that the case completeness rate is dependent on the registry`s inclusion criteria [40]. Further, studies use NISS > 15 [4, 40, 41], Injury Severity Score (ISS) ≥ 16 [42], Trauma Team Activation [16] and “all trauma patients admitted to hospital after emergency department evaluation” [15] as study inclusion criteria. This is important knowledge when comparing publications.

The current study showed almost perfect agreement (AC_1_ /AC_2_/ ICC > 0.80) for all tested variables. With high average observed agreement of 95.6% (range 76.7 − 100.0%) it also supports good results (yet, not directly comparable) for overall accuracy of 94.3% in a Finnish trauma registry study [4] and 85.8% in a Swedish study [40], and for the average rate of complete concordance of 98.0% in the Navarre Trauma Registry in Spain [43]. High agreement levels for evaluated variables in the NTR can be explained by the fact that all registrars are nurses with experience in treating trauma patients, are certified in AIS scoring by the Association for the Advancement of Automotive Medicine [44], and have completed a mandatory NTR coding course before they are licenced to code in the database. Additionally, the NTR has invested much time in developing a uniform data definition catalogue based on the Utstein template [5], iterative development of registration guidelines and user-friendly digital registration solutions with compulsory fields.

The accuracy of the AIS variable has previously been investigated in several publications, as it is a key component of the ISS and NISS (i.e. injury severity grading) [3, 16, 42], but was not explored in our study. The ISS value in the present study had an excellent agreement of ICC 0.96 (95% CI: 0.94–0.97), which is slightly better compared to previous publications by Olthof et al. (ICC 0.84) [42] and Horton et al. (ICC 0.87) [15]. The results of the ISS and NISS analyses in our paper show marked improvements compared to results in a study by Ringdal et al. (ICC 0.96 vs. ICC 0.51) [45]. Ringdal et al. found moderate to substantial (quadratic weighted Kappa: 0.66–0.96) inter-rater reliability between data coders for pre-injury ASA which was confirmed in this study by corresponding substantial to almost perfect grouping for quadratic weighted Kappa 0.67–0.88) [45]. Potential reasons for not obtaining excellent agreement for the ASA value may not only be caused by misclassification errors by the raters, but also because clinical information about pre-existing medical conditions in the trauma patient may be missing or difficult to classify in specific categories. Such coding guidelines may be insufficiently precise for use by a general rater population. Development of more concise coding guidelines may further improve the performance of this scale [45].

### Missing data

Lack of registry data caused by reduced completeness may weaken the trustworthiness, value of advice and conclusions inferred from medical registries. In a systematic review investigating missing data in trauma registries, the authors found that the majority of publications did not quantify the extent of missing data for any variables [14]. Additionally, two other publications highlighted that the extent of missing data is not well described [12, 17]. In our study, completeness of registrations was lower for “Pre-hospital SBP”, “Pre-hospital Respiratory Rate (RR)”, “In-hospital SBP” and “In-hospital RR”. This can be explained by the fact that some registrations were free text, although, numerical data were required. Those registrations were excluded from the analysis (e.g. “In-hospital RR” had 11 free text answers, those were excluded resulting in *n* = 169 for this variable, Table 3). We found a substantial amount of missing data for three continuous variables “Base excess”, “Pre-hospital SBP”, “Pre-hospital RR”. We also observed that some time variables had high percentages of missing data. These findings are consistent with findings from Ringdal et al. who also identified “Arterial Base Excess”, “Time until Normal Arterial Base Excess” and “Pre-hospital Respiratory Rate” as the variables with lowest levels of completeness [41]. Two other studies have also reported high levels of missing data for the exact same variables and in general, time variables have a higher missing rate compared to categorical variables [4, 43]. Exact values of prehospital respiratory rate are often missing in databases and are continually reported as missing in research papers. One solution is to accept categorical values of RR, which we allow in the NTR, another would be to exclude RR from the registry. Ekegren et al. points out that an outcome which should detect differences over time needs to have adequate validity (i.e. measure what it is supposed to measure), reliability (i.e. show consistency over time or similarity between raters) and responsiveness to change (i.e. ability to detect change) [46, 47]. Therefore, the efforts and time spent on registration should serve a defined purpose of improving care for the potentially severely injured patients. The questions have been raised, whether variables that consistently show high missing rates should be removed from the template or at least be reconsidered [4, 41].

Causes of missing data are likely to be multifactorial. In the study by Heinänen et al., the authors observed that in most cases, the main reason for missing data was the actual lack of documentation in the patient charts, which was particularly evident for pre-hospital documentation [4]. This does not necessarily show the quality of the registry itself, but underline the challenges in documentation of clinical processes and outcomes. Examples of this might be severely injured patients undergoing immediate lifesaving procedures when transportation is prioritized over documentation, or in cases where interventions (e.g. blood samples – Base Excess) are not performed in minor injured patients due to little clinical relevance and/or reduction of unnecessary pain/discomfort (e.g. pediatric population). A systematic review described that important trauma registry variables, such as physiological variables, are unlikely to be missing completely at random, which may be due to causes described above [14]. Methods to differentiate if procedures and/or measurements actually are performed, may give us more in-depth understanding of the actual root cause of the problem.

### Improving data quality

A frequent cause of incorrect data is often human, and methods to avoid these errors are important to make trauma registry data more reliable [4]. Increased use of structured and automated extraction of data from electronic patient records could reduce inter-individual differences. In a literature review from 2002, the authors found a 2% missing data in automatically collected versus 5% in manually collected registry data [19]. As the development in automated systems has been substantial during the last two decades, one might expect that implementation of such systems would enhance data quality considerably. Varmdal et al. suggest developing a real-time data collection system for recording stroke onset time to correct weaknesses in the data [27]. Although the technological advancements are available, implementation of such systems and simultaneously safeguarding the medical and judicial requirements, need to be aligned.

### Strengths and limitations

Assessment of data quality in the NTR has been proven to be an intense and time-consuming process. Applying this process to the whole dataset can be difficult to implement in practice in periodic audits. The current study represents an attempt to validate for the first time a large number of core variables that constitute national quality indicators of the NTR and the Utstein Trauma Template. The authors perceive the selected hospitals as representative for the broader population of the NTR. Yet, the sample size is not sufficient to perform analyses at the level of each hospital, and this can be regarded as a potential limitation of the study.

The study has the advantage of being based on a well-designed database, which with its added infrastructure (e.g. use of certified registrars, systematic quality assurance of registrations) contribute to higher quality of data being registered into the NTR. However, the possible limitation can be that we did not define EPRs as the “gold standard” required to measure the accuracy of data in terms of sensitivity and specificity as we assumed some possible level of errors associated with the process of re-entering of registrations from EPRs (likely to be minimal, though) and the data source itself. Yet, statistical measures of agreement (e.g. Cohen’s Kappa, Gwet’s AC_1_/AC_2_, ICC etc.) are usually used when verifying data against source information where the “gold standard” was not established, and high agreement between the two data sources suggests that the registry’s data elements have high validity [17, 48–50]. The methodology used in the current study has potential generalizability and is applicable to other similar medical quality registries when assessing data quality. However, one should be aware that classification and labeling of agreement coefficients into the groups is arbitrary, though, widely used when reporting the results. While having obtained high agreement levels, disagreements can still occur that are clinically unacceptable.

Our findings describe the current situation for a well-developed healthcare system in a high-income country, with a small population and a tradition with nationwide health quality registries. This implies that findings are probably more easily generalizable to high-income countries with similar traditions of nationwide health and death registries that can be linked. A comparison of data across countries with different health systems, income level and health registries may require collection of data that are less resource demanding, e.g. a set of anatomic injury descriptors with fewer codes.

## Conclusion

Core variables that compose national quality indicators in the Norwegian Trauma Registry have high agreement levels when compared with corresponding variables in electronic patient records. This indicates that the registry has accurate and valid data that can be used with confidence in research, quality improvement work and as a basis for defining public health policy. In this study, we also identified certain problematic variables related to incomplete data, which in some cases were due to poor documentation of pre-hospital values of individual patients. We should therefore scrutinize if those data are important to collect for the registry and if the Utstein criteria should be revised as well.

### Electronic supplementary material

Below is the link to the electronic supplementary material.

Additional File 1. Validated variables in the National Trauma Registry

Additional File 2. Trait distribution for nominal and ordinal variables in the National Trauma Registry

## Data Availability

Data is available upon reasonable request.

## Abbreviations

AC: Agreement coefficient
AIS: Abbreviated Injury Scale
ASA: American Society of Anesthesiologists physical status
CI: Confidence Interval
CT: Computer Tomography
EPR: Electronic Patient Record
GCS: Glasgow Coma Scale
GOS: Glasgow Outcome Score
ICC: Intraclass Correlation Coefficient
ICU: Intensive Care Unit
ISS: Injury Severity Score
LOS: Length of Stay
MOI: Mechanism of Injury
MRS: Medical Registration Solution
NISS: New Injury Severity Score
NTR: Norwegian Trauma Registry
RHA: Regional Health Authority
RR: Respiratory Rate
SBP: Systolic Blood Pressure
SD: Standard Deviation
